# Relationship Between Time to Initiation of Antiretroviral Therapy and Treatment Outcomes: A Cohort Analysis of ART Eligible Adolescents in Zimbabwe

**DOI:** 10.1097/QAI.0000000000001274

**Published:** 2016-12-15

**Authors:** Florian Vogt, Andrea M. Rehman, Katharina Kranzer, Mary Nyathi, Johan Van Griensven, Mark Dixon, Wedu Ndebele, Hilary Gunguwo, Robert Colebunders, Mbongeni Ndlovu, Tsitsi Apollo, Rashida A. Ferrand

**Affiliations:** * Institute of Tropical Medicine Antwerp, Antwerp, Belgium;; † London School of Hygiene and Tropical Medicine, London, United Kingdom;; ‡ National and Supranational Reference Laboratory, Research Centre Leibnitz, Borstel, Germany;; § Mpilo Central Hospital, Bulawayo, Zimbabwe;; ‖ National University of Science and Technology, Bulawayo, Zimbabwe;; ¶ University of Antwerp, Antwerp, Belgium;; # Ministry of Health and Child Welfare, Harare, Zimbabwe;; ** University of Zimbabwe, Harare, Zimbabwe; and; †† Biomedical Research and Training Institute, Harare, Zimbabwe.

**Keywords:** ART initiation, adolescents, eligibility, mortality, loss to follow-up, Zimbabwe

## Abstract

**Background:**

Age-specific retention challenges make antiretroviral therapy (ART) initiation in adolescents difficult, often requiring a lengthy preparation process. This needs to be balanced against the benefits of starting treatment quickly. The optimal time to initiation duration in adolescents is currently unknown.

**Objective:**

To assess the effect of time to ART initiation on mortality and loss to follow-up (LTFU) among treatment eligible adolescents.

**Methods:**

We conducted a retrospective cohort analysis among 1499 ART eligible adolescents aged ≥10 to <19 years registered in a public sector HIV program in Bulawayo, Zimbabwe, between 2004 and 2011. Hazard ratios (HR) for mortality and LTFU were calculated for different time to ART durations using multivariate Cox regression models.

**Results:**

Median follow-up duration was 1.6 years. Mortality HRs of patients who initiated at 0 to ≤7 days, >14 days to ≤1 month, >1 to ≤2 months, >2 months, and before initiation were 1.59, 1.19, 1.56, 1.08, and 0.94, respectively, compared with the reference group of >7 to ≤14 days. LTFU HRs were 1.02, 1.07, 0.85, 0.97, and 3.96, respectively. Among patients not on ART, 88% of deaths and 85% of LTFU occurred during the first 3 months after becoming ART eligible, but only 37% and 29% among adolescents on ART, respectively.

**Conclusions:**

Neither mortality or LTFU was associated with varying time to ART. The initiation process can be tailored to the adolescents' needs and individual life situations without risking to increase poor treatment outcomes. Early mortality was high despite rapid ART initiation, calling for earlier rather than faster initiation through HIV testing scale-up.

## Abstract 2

Supplemental Digital Content is Available in the Text.

## INTRODUCTION

Antiretroviral therapy (ART) scale-up has reduced HIV-related deaths substantially.^1^ Adolescents remain the only age group where HIV-associated mortality is still rising, mainly because of delayed diagnosis and high attrition, and in particular around the time of ART initiation.^2–7^ Large cohorts of vertically infected infants born during the peak of the epidemic are now growing into adulthood without ever having been diagnosed or treated.^8–10^ This adolescent HIV epidemic of “slow progressors”^11–13^ has been unfolding largely unnoticed for many years.^14–16^ Only recently have adolescents been receiving more attention on the global HIV agenda.^17–20^

Currently, multiple preparatory counseling and education sessions stretching over several weeks are considered necessary before treatment start to ensure long-term retention and adherence.^21–24^ Adolescents living with HIV face particularly complex issues such as emerging sexuality, peer influence, stigma, delayed disclosure of diagnosis, and consent legislation, all of which complicates retention and adherence to ART.^2,19,25–28^ Addressing these issues takes time and can result in delaying ART initiation even if clinical treatment eligibility criteria are met.^29^ This needs to be balanced against the well-established life-saving effects of rapid treatment initiation.^30^ Also, a lengthy pre-initiation preparatory phase can have negative effects on access to and linkage of care.^24,31–37^

Existing research on ART initiation in adolescents revolves mostly around comparing clinical outcomes before and after ART initiation,^6,38^ or between adolescents and adults,^3,5,7,39,40^ or around determining the best clinical time point to start treatment.^30,41^ The period between becoming ART eligible and starting treatment, the so-called “third stage” of pre-ART care,^42^ is often overlooked,^43^ which is particularly concerning as guidelines now recommend treatment for all HIV-infected individuals regardless of age or immune status.^44^ For the ART initiation process in adolescents, it is currently not known how fast is “fast enough but not too fast” to keep both mortality and loss to follow-up (LTFU) before and after ART initiation at a minimum.^20^ A better understanding of the effect of varying time to ART initiation in long-term infected adolescents would help clinicians decide as to how much the initiation process can be adapted to individual patient needs without compromising clinical outcomes.

We investigated treatment initiation patterns and related patient outcomes, and in particular the effect of time to ART initiation on mortality and LTFU among treatment eligible adolescents aged ≥10 to <19 years registered in a public sector HIV care service in Bulawayo, Zimbabwe.

## METHODS

### Setting

Bulawayo is the second biggest city in Zimbabwe, a country that has been experiencing an early-onset severe generalized HIV epidemic.^45,46^ HIV prevalence in Zimbabwe peaked in 1997, reaching 27% in the general population,^47^ and 15% and 8% among women and men aged 15–24 years, respectively.^48^ During the late 1990s, HIV-related mortality increased 5-fold in Zimbabwe, peaking to 11 per 1000 persons per year in 2002, and has been declining gradually since then.^45^

Mpilo Central Hospital, the biggest public health facility in Bulawayo, opened an HIV clinic in 2004 in collaboration with the non-governmental organization Doctors Without Borders (Médecins Sans Frontières), and was one of the first facilities nationwide to provide ART. Patients entered the HIV program of the Mpilo ART clinic usually after testing positive at one of the outpatient or inpatient departments of the hospital, in the private sector, or at stand-alone voluntary counseling and testing facilities. ART was initiated by doctors with follow-up care being provided by nurses trained in HIV/AIDS management. ART eligibility criteria during the study period were a cluster of differentiation type 4 (CD4) cell count of ≤200 cells per microliter, or World Health Organization (WHO) stage 3 or 4 HIV disease.^49,50^ ART eligibility was routinely assessed at each patient visit. In Zimbabwe, as in many other countries in Sub-Saharan Africa (SSA), initiation of ART in eligible patients is recommended following 2 to 3 visits for treatment preparedness counseling.^39,51,52^ Systematic tracing of defaulting ART patients, defined as having missed a scheduled appointment by more than 2 months, was carried out by community volunteers through home visits and telephone calls. Mpilo hospital remained the only provider of ART initiation services for adolescents in the wider Bulawayo area throughout the study period.

### Study Design and Population

We conducted a cohort analysis using routinely collected data from the HIV program at Mpilo Hospital in Bulawayo, Zimbabwe. All data collection had ended before this research idea was conceived. Records from all patients aged ≥10 to <19 years with confirmed ART eligibility between February 2004 and September 2011 were considered eligible for this analysis.

### Data Management and Analysis

Clinical data were entered from paper-based hospital charts into an electronic database (FUCHIA, Epicentre, Paris), and updated after each patient visit as part of routine program activities. Patient outcomes were obtained on an ongoing basis from defaulter tracing activities, community reporting, and death register reviews, and entered into the FUCHIA database.

Patient outcomes were defined as died, retained (being alive and in care with ≤3 months since last recorded visit), LTFU (outcome unknown with >3 months since last recorded visit; time of LTFU was set at 30 days after last recorded visit), and transferred out. Time to ART initiation was defined as time elapsed between becoming ART eligible and initiating ART, and was grouped into 5 categories (0 to ≤7 days, >7 to ≤14 days, >14 days to ≤1 month, >1 to ≤2 months, and >2 months). Person-time accruing before initiation was categorized separately. Available covariates were sex, age (≥10 to <15 or ≥15 to <19 years), WHO staging (clinical stage ≤2 or >2), CD4 count (0 to ≤200 or >200 cells/μL) and calendar year of ART eligibility.

The database was censored at 24 months after becoming ART eligible. Patients who had their first visit <30 days before the end of the study period (September 1, 2011) were excluded. ART initiation and incidence of death, LTFU, and transferred out by treatment status were calculated for different follow-up intervals. Cumulative hazards were plotted for mortality and LTFU to assess differences by time to ART initiation. Crude and adjusted rates and hazard ratios (HR) using Cox regression analysis, including 95% confidence intervals (95% CI) and *P*-values from likelihood ratio tests, were calculated. Departure from the proportional hazard assumption was evaluated graphically using logarithmic plots, as well as formally assessed by testing for changes in HR over time using likelihood ratio tests. Time-dependent exposure assignment based on the Mantel-Byar approach was used to account for immortal time bias.^53^ Sex, age, WHO staging, and calendar year of ART eligibility were considered *a priori* confounding factors and adjusted for in the multivariate models. CD4 count was not included because of the high proportion of missing values. Effect modification for calendar year was explored for both mortality and LTFU. Statistical analyses were conducted using the STATA v.14 software (StataCorp, College Station, TX).

### Ethics

Data collection was covered by a memorandum of understanding between Médecins Sans Frontières and the Zimbabwean Ministry of Health. Permissions to use these data for analysis and publication were obtained from both entities. As only anonymized data were used, and no intervention or patient contact was made for research purposes, the issue of informed consent did not apply. The requirement for ethical approval was waived by the Mpilo Hospital Institutional Review Board, Bulawayo, and by the National Medical Research Council of Zimbabwe, Harare.

## RESULTS

Of the 2184 adolescents enrolled into the HIV program, 1506 (69%) met ART eligibility criteria during the study period. Of these, 20% (307/1506) met CD4 and WHO criteria, 13% (195/1506) met CD4 but not WHO criteria, 17% (256/1506) met WHO but not CD4 criteria, and 50% (748/1506) met WHO criteria with CD4 results missing. Because of missing CD4 results, ART eligibility could not be confirmed for 17% (364/2184) of enrolled adolescents who did not meet the WHO criteria (see Table, Supplemental Digital Content 1, http://links.lww.com/QAI/A961, showing patient characteristics by ART eligibility status). Seven of the 1506 ART eligible patients (0.5%) had to be excluded from analysis because of insufficient follow-up time or missing data (see Figure, Supplemental Digital Content 2, http://links.lww.com/QAI/A961, showing patient record selection for analysis). The remaining 1499 patients provided a total of 3097 person-years of follow-up (median, 1.6 years; interquartile range [IQR] 0.7–3.2). Median time to ART initiation was 17 days (IQR, 9–42). Ten percent (150/1499) of patients did not start ART. Median age was 13.2 years (IQR, 11.3–15.2), and 52% (773/1499) of the adolescents were women. Most patients (87%; 1305/1499) had WHO stage 3 or 4 HIV disease. The median CD4 cell count was 145 cells per microliter (IQR, 44–267) for the 754 patients (50%) with available test result (Table 1).

**TABLE 1.**
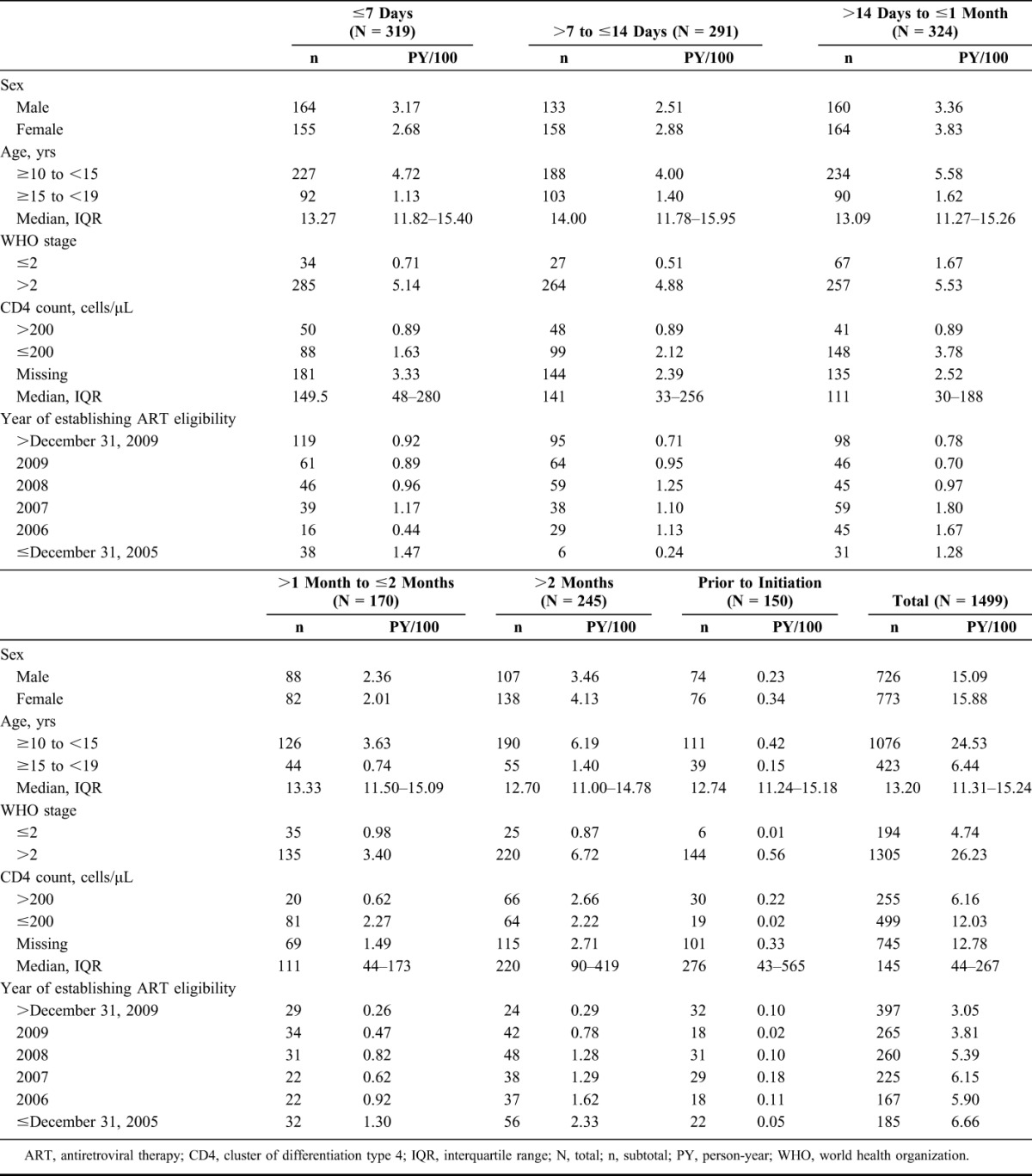
Patient Characteristics at Baseline by Time to ART Initiation

**FIGURE 1.**
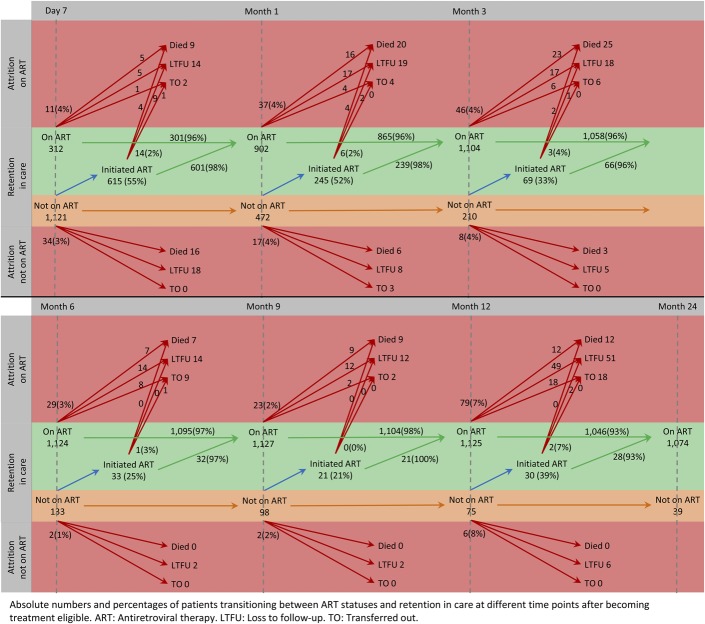
ART status transitions and patient outcomes after becoming ART eligible.

At 24 months after becoming ART eligible, 72% of patients (1074/1499) were retained and on ART. Mortality and LTFU was 6% (84/1332) and 10% (133/1332) among patients on ART, and 15% (25/167) and 59% (98/167) among patients not on ART, respectively. Half of all deaths (49%; 53/109) and LTFU (52%; 121/231) occurred within the first 3 months after becoming ART eligible. Among patients not on ART, the most deaths (88%; 22/25) and LTFU (85%; 83/98) occurred during this period, but only 37% (31/84) and 29% (38/133) among adolescents on ART, respectively (Table 2). New ART initiations decreased with prolonged time elapsing after becoming ART eligible. However, those who initiated ART slower had no worse outcomes than those initiating fast, or than those being on ART already for longer time (Fig. 1).

**TABLE 2.**
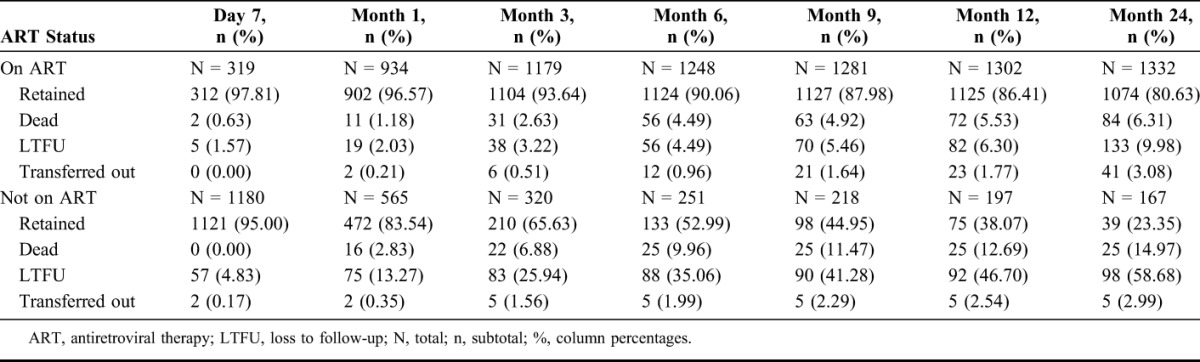
Patient Outcomes After Becoming ART Eligible by ART Status

**FIGURE 2.**
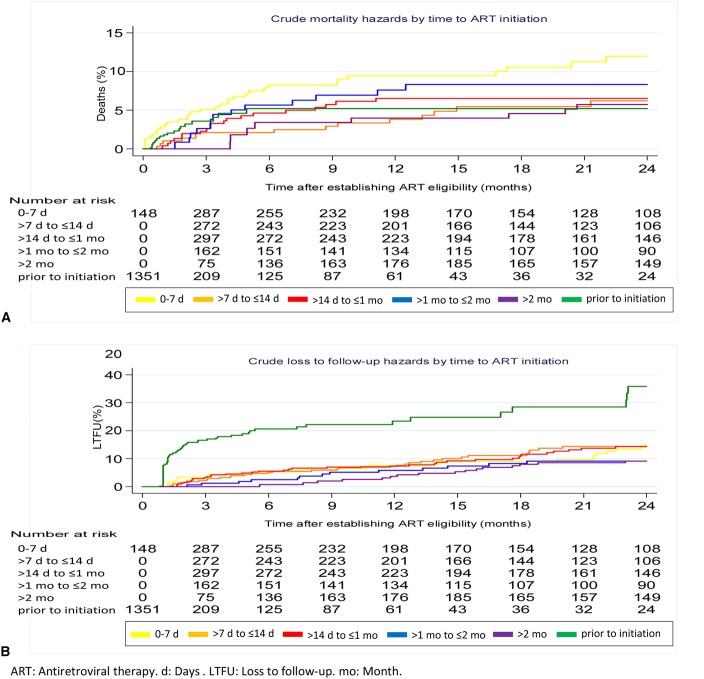
Cumulative hazards for mortality (A) and for loss to follow-up (B) by time to ART initiation.

The crude mortality rate during the 24 months after becoming ART eligible was 5.46/100 person-years (95% CI: 4.53 to 6.59). The corresponding LTFU rate was 11.32/100 person-years (95% CI: 9.94 to 12.90). Stratified by time to ART initiation, cumulative hazards for mortality were highest in patients with fastest initiation (0 to ≤7 days). LTFU was highest in patients before ART initiation. However, no clear pattern emerged for mortality or LTFU with increasing time to ART duration (Fig. 2). After adjusting for sex, age, WHO stage, and calendar year, HRs for mortality were 1.59 (95% CI: 0.83 to 3.04), 1.19 (95% CI: 0.59 to 2.40), 1.56 (95% CI: 0.72 to 3.41), 1.08 (95% CI: 0.44 to 2.71), and 0.94 (95% CI: 0.46 to 1.92) for patients with ART initiation at 0 to ≤7 days, >14 days to ≤1 month, >1 to ≤2 months, >2 months, and before initiation, respectively, using patients with ART initiation at >7 to ≤14 days as reference group. The corresponding adjusted HR for LTFU were 1.02 (95% CI: 0.62 to 1.67), 1.07 (95% CI: 0.66 to 1.73), 0.85 (95% CI: 0.44 to 1.64), 0.97 (95% CI: 0.52 to 1.81), and 3.96 (95% CI: 2.60 to 6.04) (Table 3). There was no evidence of effect modification by calendar year for either mortality or LTFU.

**TABLE 3.**
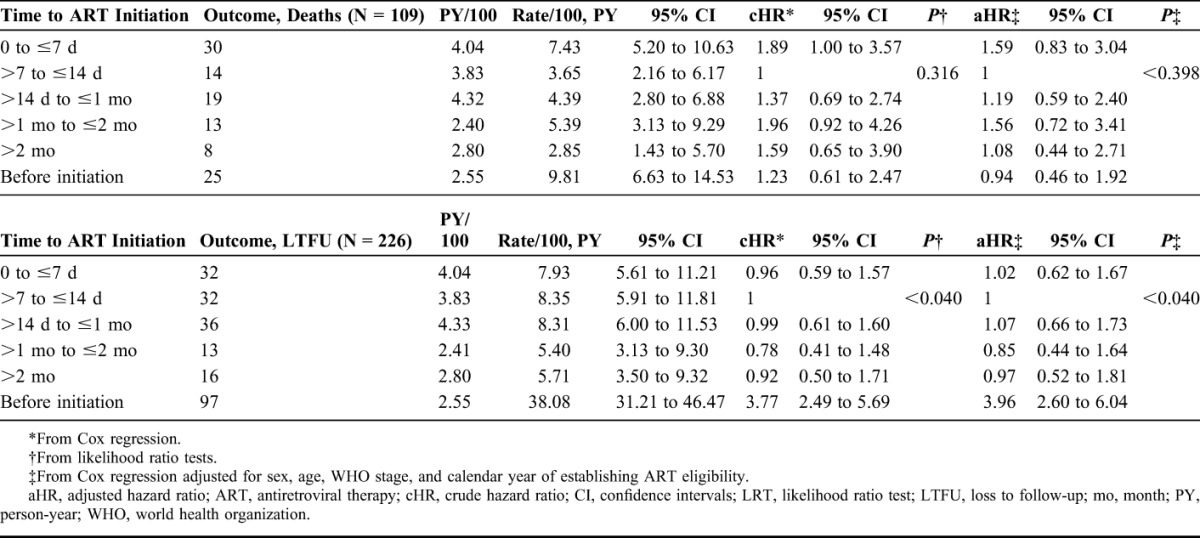
Crude and Adjusted Rates and Hazard Ratios for Mortality and for Loss to Follow-up by Time to ART Initiation

## DISCUSSION

To our knowledge, this is the first assessment of ART initiation dynamics and related patient outcomes, and the effect of varying time to ART initiation on mortality and LTFU among treatment eligible adolescents. Retention was high overall, and most patients started ART rapidly. We found high mortality and LTFU shortly after becoming eligible, especially among adolescents not on ART, but there was no evidence for differences in mortality or LTFU among adolescents with varying time to ART duration.

High mortality and LTFU around the time of becoming ART eligible has also been found elsewhere across different settings and patient groups, in particular among patients not initiated on ART.^6,33,54–57^ Considering that many of those coded as LTFU are like to have actually died,^58,59^ mortality among our patients is probably even higher than reported in the data. We attribute the slightly increased mortality among patients initiated at 0 to ≤7 days in our analysis to selection bias caused by health care workers prioritizing sicker patients, who had a higher probability of dying despite fast ART initiation, over less sick patients. This effect probably outweighed the benefits of rapid ART initiation on survival in these patients. The fact that we did not find elevated LTFU in patients who initiated rapidly suggests that, contrary to current understanding,^21–24^ a long preparatory process is not needed to keep post-initiation LTFU low.

Our findings are supported by another routine program evaluation from South Africa, which found high ART uptake, good adherence, and no evidence for increased post-initiation LTFU after introducing a comprised preparation schedule for accelerated treatment start within 1 week.^60^ Similarly encouraging evidence is emerging from a randomized trial in Haiti, where preliminary results suggest better retention and lower mortality among patients initiated at the same day of establishing ART eligibility compared with patients who initiated after 3 weekly preparatory visits.^58^ Another recently completed cluster-randomized trial in Uganda using a multicomponent intervention primarily targeting health care workers to accelerate ART initiation, including the option to start ART at the same day of HIV diagnosis, found better adherence among rapidly initiated patients, and no difference in LTFU and mortality compared with standard of care.^59^ However, some evidence for increased post-initiation loss to care was found in a small randomized trial from South Africa, in which patients in the intervention arm were offered same-day initiation compared with 3 or 4 preparation visits over up to 4 weeks in standard of care.^61^ Interestingly, considerable flexibility was granted in both arms in this trial, which allowed patients in the intervention arm to delay up to 30 days, and patients in the comparison group to initiate after only 1 week if considered necessary. Notably, all mentioned studies excluded patients below the age of 18 years. Findings can hence not be readily applied to adolescents. There is still no evidence that patients actually benefit from the currently prevailing initiation model with multiple obligatory preparation visits.^32^ Ultimately, much still needs to be learnt about how to best initiate ART in adolescents, in particular regarding the number and timing of preparatory sessions once ART eligibility criteria are met.^43^ Patients' individual readiness rather than adherence to rigid counseling schedules should remain the guiding principal.^62^ However, providing individualized care will become increasingly difficult in SSA as the number of eligible patients continues to rise with eligibility criteria being steadily broadened.

ART initiation is a crucial step in the continuum of HIV care to reduce mortality.^63–66^ In our study, early mortality on ART was high despite the relatively short median time to ART initiation of 17 days. Also, CD4 levels at the time of becoming ART eligible were relatively low in our cohort. This makes it unlikely that shortening the time to ART duration further will have substantial effects on mortality. Instead, scale-up of HIV testing coupled with effective pre-ART follow-up, broad eligibility criteria, and better access to care, thereby initiating ART earlier in a larger number of infected adolescents, is needed to reduce mortality. Although eligibility continues to be widened in Zimbabwe and globally,^44,52^ provision and uptake of testing services remains challenging.^67^

Our analysis demonstrates important pitfalls in ART outcomes research. Status transitions between not yet being ART eligible, being eligible but not yet on ART, and being on ART, and how these dynamics relate to patient outcomes are difficult to disentangle. Mere pre- vs post-initiation outcome comparisons to assess the effect of ART, as commonly done, are misleading because the varying durations between eligibility and ART initiation cannot be accounted for. Existing research rarely considers the transient nature of care during this phase, with eligible patients starting and stopping treatment at varying time points, thereby leading to a skewed understanding of the effect of ART initiation on treatment outcomes.^20^ In addition, risk comparisons based on survival analyses are inevitably hampered by bias and competing risks of LTFU and death. Allowing patients to switch treatment status, and to present percentages of cumulative and incident outcomes accordingly, as done in this analysis (Table 2 and Fig. 1), provides a more realistic picture of this crucial phase of HIV care.

Other strengths of this study are its big sample size and standardized data collection by trained encoders into a dedicated database with regular systematic data quality checks. By using programmatic data originating from a routine care setting in a public sector facility, our findings are more realistic of real-life conditions in HIV programs from resource-limited settings than evidence from controlled trial environments.

This research is subject to the usual limitations inherent to analyses using existing program data, most notably missing data. First, ART eligibility could not be confirmed for 364/2184 (17%) of all enrolled patients. Although their age and sex distribution was similar to included patients, fewer deaths occurred in this group (see Table, Supplemental Digital Content 1, http://links.lww.com/QAI/A961, showing patient characteristics by ART eligibility status), which might have biased our results. Second, CD4 results could not be included in the adjusted model because of missing data. Third, because follow-up and documentation of patients not yet ART eligible was patchy, only data after becoming ART eligible could be used. Information about the time point and mode of HIV infection, and regular eligibility status updates of patients before becoming ART eligible would have allowed to assess the evolution of the immune response and hence the clinical matureness of our cohort at the time of becoming ART eligible. Fourth, the number of preparatory appointments each patient actually attended before ART initiation was not known. Fifth, reasons for deliberate delays in ART initiation were not captured during program implementation. For example, we could not account for clinical conditions such as tuberculosis or cryptococcal infection, or particular psychosocial circumstances that demanded deferred ART initiation. Last, many SSA countries have now changed ART initiation thresholds from 200 to 350 or 500 CD4 cells per microliter,^44,52^ or are moving toward a general test and treat approach. However, a substantial number of HIV-infected adolescents today are perinatally infected long-term survivors,^8–10^ thus having a delayed diagnosis by definition, and start ART with low CD4 levels because of the persisting testing gap. Therefore, the question of how fast to initiate such patients on diagnosis remains pertinent. In addition, the complexities of ART initiation in this age group have not changed. The upcoming test-all-treat-all era will increase the need for preparatory counseling massively, and hence make it even more crucial to find efficient yet sustainable ways to prepare patients for ART. However, our results should be treated with caution when applied to patients with high CD4 levels who were not eligible for ART during the study period. Numbers of patients with high CD4 counts are likely to increase in the future through the treat-all approach.

In summary, we found high mortality during the first 3 months following ART eligibility despite fast treatment initiation. Starting ART earlier through broader eligibility criteria and scale-up of HIV testing in adolescents, rather than faster initiation, is needed to reduce these deaths. We found no association between rapid treatment initiation and increased postinitiation LTFU. This adds to the mounting evidence that health care providers and care givers should be given considerable flexibility to tailor the initiation process to adolescents' individual needs and life situations during this period.

## Supplementary Material

SUPPLEMENTARY MATERIAL
