# Supplementary material for: Relationship Between Time to Initiation of Antiretroviral Therapy and Treatment Outcomes: A Cohort Analysis of ART Eligible Adolescents in Zimbabwe
Source: J Acquir Immune Defic Syndr. 2016 Dec 15;74(4):390–8. doi: 10.1097/QAI.0000000000001274 (PMC5321111; doi:10.1097/QAI.0000000000001274)
Supplement: SUPPLEMENTARY MATERIAL [file qai-74-390-s001.docx]

**Supplemental Digital Content 1: Patient characteristics by ART eligibility status.**

|  | **ART eligible**  (N=1,506) | | **Not ART**  **eligible**  (N=314) | | **ART eligibility**  **unknown**  (N=364) | | **Total**  (N=2,184) | |
| --- | --- | --- | --- | --- | --- | --- | --- | --- |
| **Patient characteristics** | n | % | n | % | n | % | n | % |
| **Sex** |  |  |  |  |  |  |  |  |
| male | 730 | 48.47 | 134 | 42.68 | 163 | 44.78 | 1,027 | 47.02 |
| female | 774 | 51.39 | 177 | 56.37 | 201 | 55.22 | 1,152 | 52.75 |
| *missing* | *2* | *0.13* | *3* | *0.96* | *0* | *0.00* | *5* | *0.23* |
| **Age** (years) |  |  |  |  |  |  |  |  |
| ≥10-<15 | 1,078 | 71.58 | 231 | 73.57 | 269 | 73.90 | 1,578 | 72.25 |
| ≥15-<19 | 428 | 28.42 | 83 | 26.43 | 95 | 26.10 | 606 | 27.75 |
| Median (IQR) | 13.23 | 11.45- 15.30 | 12.93 | 11.29- 15.05 | 13.07 | 11.29-15.09 | 13.15 | 11.39-15.22 |
| **WHO stage** |  |  |  |  |  |  |  |  |
| ≤2 | 195 | 12.95 | 314 | 100.00 | 364 | 100.00 | 873 | 39.97 |
| >2 | 1,311 | 87.05 | 0 | 0.00 | 0 | 0.00 | 1,311 | 60.03 |
| **CD4 count** (cells/µL) |  |  |  |  |  |  |  |  |
| >200 | 256 | 17.00 | 314 | 100.00 | 0 | 0.00 | 570 | 26.10 |
| ≤200 | 502 | 33.33 | 0 | 0.00 | 0 | 0.00 | 502 | 22.99 |
| *missing* | *748* | *49.67* | *0* | *0.00* | *364* | *100.00* | *1,112* | *50.92* |
| Median (IQR) | 145.5 | 44-265 | 400 | 283-533 | - | - | 218.5 | 95-389.5 |
| **Year of establishing ART eligibility** |  |  |  |  |  |  |  |  |
| >31DEC2009 | 403 | 26.76 | 140 | 44.59 | 103 | 28.30 | 646 | 29.58 |
| 2009 | 265 | 17.60 | 20 | 6.37 | 71 | 19.51 | 356 | 16.30 |
| 2008 | 260 | 17.26 | 20 | 6.37 | 116 | 31.87 | 396 | 18.13 |
| 2007 | 225 | 14.94 | 50 | 15.92 | 46 | 12.64 | 321 | 14.70 |
| 2006 | 168 | 11.16 | 36 | 11.46 | 11 | 3.02 | 215 | 9.84 |
| ≤31DEC2005 | 185 | 12.28 | 48 | 15.29 | 17 | 4.67 | 250 | 11.45 |
| **Outcome** |  |  |  |  |  |  |  |  |
| Retained | 1,012 | 67.20 | 238 | 75.80 | 237 | 64.11 | 1,487 | 68.09 |
| Dead | 133 | 8.83 | 5 | 1.59 | 15 | 4.12 | 153 | 7.01 |
| LTFU | 300 | 19.92 | 59 | 18.82 | 93 | 25.55 | 452 | 20.70 |
| Transferred out | 61 | 4.05 | 12 | 3.79 | 19 | 5.22 | 92 | 4.21 |

ART: Antiretroviral therapy. CD4: Cluster of differentiation type 4. DEC: December. IQR: Inter-quartile range.. N: Total. n: Sub-total. WHO: World Health Organization. %: Column percentages.

**Supplemental Digital Content 2: Patient record selection for analysis.**

**Excluded**

**314** patients not ART eligible

**364** patients with unknown ART eligibility

**Excluded**

**5** patients with first visit date ≤30 days prior to database censor date (4 started ART)

**2** patients with missing data for sex (1 started ART)

**Total**

**2,184** patients aged ≥10-<19 enrolled in the HIV programme in Bulawayo, Zimbabwe between February 2004 and September 2011

**ART eligible**

**1,506** patients

**Analysed**

**1,499** patients

ART: Antiretroviral therapy. HIV: Human immunodeficiency virus. .
